# Tracking AC Electric Stimulation–Induced Persistent Locomotion Behavior in the Nematode *Caenorhabditis elegans*

**DOI:** 10.21769/BioProtoc.5733

**Published:** 2026-07-05

**Authors:** Mina Nakasone, Shuma Hanabaru, Kotono Sekizawa, Shiho Namba, Ryoga Suzuki, Koutarou D Kimura

**Affiliations:** 1 Graduate School of Science, Nagoya City University, Nagoya, Japan; 2 School of Science, Nagoya City University, Nagoya, Japan

**Keywords:** *C. elegans*, AC electric stimulation, Persistent behavior, Video tracking, Genetic analysis

## Abstract

Persistent neural activity underlies fundamental brain functions such as memory, decision-making, and emotion. Despite its importance, experimental paradigms that enable quantitative analysis of persistent behavioral responses remain limited. Here, we describe a protocol to induce and measure a persistent locomotor response by applying a brief alternating current (AC) electric stimulus to the nematode *Caenorhabditis elegans*. This method reliably evokes a prolonged increase in locomotion speed that persists for minutes after stimulus termination and can be quantified by video tracking. Because *C. elegans* has a fully mapped connectome and is amenable to genetic and neurophysiological manipulation, this protocol provides a useful platform for dissecting the molecular and neural mechanisms underlying persistent behavioral responses. Electrically induced persistent locomotion serves as a simple, robust, and quantifiable behavioral readout for studying the regulation of neural persistence in vivo.

Key features

• A brief AC electric stimulus reliably induces a persistent locomotor response in *C. elegans*.

• The assay uses a simple setup and provides reproducible behavioral data.

• Video-tracking analysis enables quantitative measurement of locomotion speed and response persistence.

• This protocol enables quantitative comparisons and analyses of behavioral changes evoked by stimulus characteristics and/or genetic mutations.

## Graphical overview


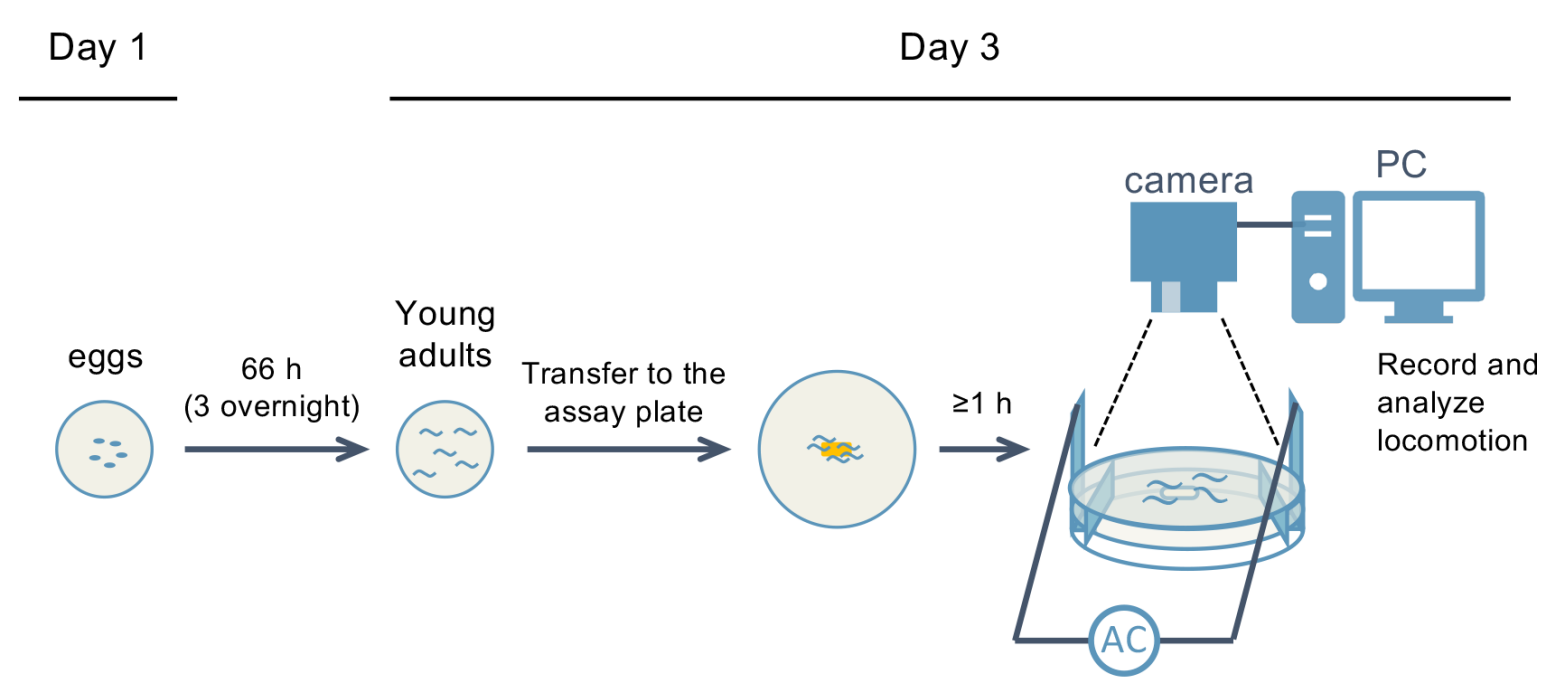


## Background

The nervous system not only responds transiently to external stimuli but can maintain activity after stimulus termination. Such persistent neural activity is thought to underlie fundamental brain functions, including memory, decision-making, and emotion [1,2]. At the behavioral level, this neural persistence can be observed as sustained changes in locomotion or other responses following brief stimulation. Quantitative analysis of these persistent behaviors provides an important entry point for investigating the neural mechanisms supporting persistent brain states. However, experimental paradigms that reliably and quantitatively induce persistent behavioral responses remain limited.

The nematode *Caenorhabditis elegans* is a powerful model organism for analyzing neural mechanisms due to its fully mapped connectome and genetic accessibility [3]. Well-established behavioral assays in *C. elegans*, such as chemotaxis and thermotaxis, have been widely used to study sensory-driven locomotor responses [4]. However, these paradigms typically rely on continuous or long-lasting external stimuli, making it difficult to untangle whether the observed behavioral persistence reflects intrinsic neural mechanisms or is simply maintained by ongoing stimulation. In contrast, a brief alternating current (AC) electric stimulation provides a simple and highly reproducible method to deliver precisely controlled stimuli [5].

In this protocol, we describe a method to induce persistent locomotor behavior in *C. elegans* using a brief AC electric stimulus and to quantitatively analyze its temporal dynamics. This assay provides behavioral data with high reproducibility and offers a platform for investigating the regulation of persistent behavioral states.

## Materials and reagents

**Biological materials**

1. *Caenorhabditis elegans* wild-type strain N2 (Caenorhabditis Genetics Center)

2. *Escherichia coli* OP50 (Caenorhabditis Genetics Center)

**Reagents**

1. NaCl (FUJIFILM Wako, catalog number: 191-01665)

2. Bacto^TM^ Peptone (Gibco, catalog number: 211677)

3. Agar (FUJIFILM Wako, catalog number: 010-08725)

4. Cholesterol (FUJIFILM Wako, catalog number: 034-03002)

5. Ethanol (99.5%) (FUJIFILM Wako, catalog number: 057-00456)

6. CaCl_2_·2H_2_O (FUJIFILM Wako, catalog number: 038-12775)

7. MgSO_4_·7H_2_O (FUJIFILM Wako, catalog number: 131-00405)

8. KH_2_PO_4_ (FUJIFILM Wako, catalog number: 169-04245)

9. K_2_HPO_4_ (FUJIFILM Wako, catalog number: 164-04295)

10. Streptomycin sulfate (FUJIFILM Wako, catalog number: 194-08512)

11. LB broth powder (BD, catalog number: 244620)

**Solutions**

1. Assay plate (see Recipes)

2. Nematode growth media (NGM) buffer (see Recipes)

3. 0.5% cholesterol (see Recipes)

4. 1 M CaCl_2_ (see Recipes)

5. 1 M MgSO_4_ (see Recipes)

6. 1 M KPO_4_ buffer (pH 6.0) (see Recipes)

7. 10 mg/mL streptomycin solution (see Recipes)

8. LB liquid medium (see Recipes)

**Recipes**

**1. Assay plate**

**Table:** 

Reagent	Final concentration	Quantity or volume
NaCl	3 g/L	1.5 g
Bacto peptone	2.5 g/L	1.25 g
Agar	17 g/L	8.5 g
0.5% cholesterol	5 μg/mL	0.5 mL
CaCl_2_ (1 M)	1 mM	0.5 mL
MgSO_4_ (1 M)	1 mM	0.5 mL
KPO_4_ buffer (1 M, pH 6.0)	25 mM	12.5 mL
dH_2_O	—	up to 500 mL
Total	n/a	500 mL

Prepare the assay plates 2–3 days before the assay.

Mix dH_2_O, NaCl, Bacto peptone, agar, and 0.5% cholesterol, autoclave the mixture, and allow it to cool to 50 °C. After cooling, add CaCl_2_, MgSO_4_, and KPO_4_ buffer and mix thoroughly on a hot magnetic stirrer. Dispense 16 mL of the medium into each 90 mm Petri dish. Store at room temperature.

**2. NGM buffer**

**Table:** 

Reagent	Final concentration	Quantity or volume
NaCl	3 g/L	0.3 g
Bacto peptone	2.5 g/L	0.25 g
0.5% cholesterol	5 μg/mL	0.1 mL
CaCl_2_ (1 M)	1 mM	0.1 mL
MgSO_4_ (1 M)	1 mM	0.1 mL
KPO_4_ buffer (1 M, pH 6.0)	25 mM	2.5 mL
dH_2_O	—	up to 100 mL
Total	n/a	100 mL

Mix dH_2_O, NaCl, Bacto peptone, and 0.5% cholesterol, autoclave the mixture, and allow it to cool to room temperature. After cooling, add CaCl_2_, MgSO_4_, and KPO_4_ buffer and mix thoroughly. Dispense 10 mL aliquots into sterile 15 mL tubes and store at -20 °C.

**3. 0.5% cholesterol**

**Table:** 

Reagent	Final concentration	Quantity or volume
Cholesterol	0.5%	0.5 g
EtOH (99.5%)	—	100 mL
Total	n/a	100 mL

Mix EtOH and cholesterol in a 100 mL bottle and dissolve completely with stirring. Store at room temperature.

**4. 1 M CaCl_2_**

**Table:** 

Reagent	Final concentration	Quantity or volume
CaCl_2_·2H_2_O	1 M	14.70 g
dH_2_O	—	up to 100 mL
Total	n/a	100 mL

Mix dH_2_O and CaCl_2_·2H_2_O, autoclave the mixture, and allow it to cool to room temperature. After cooling, store at room temperature.

**5. 1 M MgSO_4_**

**Table:** 

Reagent	Final concentration	Quantity or volume
MgSO_4_·7H_2_O	1 M	24.64 g
dH_2_O	—	up to 100 mL
Total	n/a	100 mL

Mix dH_2_O and MgSO_4_·7H_2_O, autoclave the mixture, and allow it to cool to room temperature. After cooling, store at room temperature.

**6. 1 M KPO_4_ buffer**

**Table:** 

Reagent	Final concentration	Quantity or volume
KH_2_PO_4_	0.80 M	108.3 g
K_2_HPO_4_	0.20 M	35.6 g
dH_2_O	—	up to 1 L
Total	1 M	1 L

Mix dH_2_O, KH_2_PO_4_, and K_2_HPO_4_, autoclave the mixture, and allow it to cool to room temperature. After cooling, store at room temperature.

**7. 10 mg/mL streptomycin solution**

**Table:** 

Reagent	Final concentration	Quantity or volume
Streptomycin sulfate	n/a	10 mg
dH_2_O	—	1 mL

Mix dH_2_O and streptomycin sulfate. Store at -20 °C.

**8. LB liquid medium**

**Table:** 

Reagent	Final concentration	Quantity or volume
LB broth powder	2.5%	25 g
dH_2_O	—	up to 1 L
Total	2.5%	1 L

Mix dH_2_O and LB broth powder and dissolve completely with a magnetic stirrer. Dispense 100 mL aliquots into 100 mL bottles or 500 mL flasks and autoclave them. After cooling, store at room temperature.

**Laboratory supplies**

1. 90 mm sterile Petri dishes (IWAKI, catalog number: SH90-15)

2. 50 mL centrifuge tubes (Greiner Bio-One, catalog number: 227261)

3. 1.5 mL microcentrifuge tubes (SARSTEDT, catalog number: 72.41152.002)

4. 15 mL tubes (Thermo Fisher Scientific, catalog number: 339650)

5. Sterile 200 μL Pipette tips (QSP, catalog number: T090RLS-Q)

6. Kimwipes (NIPPON PAPER CRECIA, catalog number: S-200)

7. Parafilm (Parafilm, catalog number: PM996)

8. Copper plates (Hikari, catalog number: MC332)

9. Weighing dish (BIO-BIK, catalog number: AS-DL)

## Equipment

1. Autoclave (TOMY, model: LBS-325)

2. Hot magnetic stirrer (AS ONE, model: LSH-1D)

3. Cool incubator (PHC, model: MIR-154; MIR-254-PJ)

4. Dry heat sterilizer (ADVANTEC, model: STN620DD)

5. Refrigerated centrifuge (TOMY, model: MX-301)

6. Shaking incubator (BioShaker, model: BR-43FM)

7. Stereo dissecting microscope (OLYMPUS, model: SZX7)

8. Aspirator (AS ONE, model: GAS-1)

9. Arbitrary waveform generator (Function generator) (AS ONE, model: AWG1005)

10. Amplifier (KIKUSUI, model: PCR 500MA)

11. Analog interface (KIKUSUI, model: EX08-PCR-MA)

12. Oscilloscope (TEXIO, model: DCS-1054B)

13. Multimeter (Sanwa, model: PC720M)

14. USB optical communication unit (Sanwa, model: KB-USB7)

15. LED power supply (CCS, model: PD2-3012)

16. Ring LED light source (CCS, model: LDR2-170RD-LA)

17. Camera (The Imaging Source, model: DMK72AUC02)

18. Lens (KOWA, model: LM16HC/LM16JC5M2)

19. Desktop PC (Dell, model: Vostro 260s)

20. Laptop PC (Dell, model: Latitude 3540)

21. Lead wire with alligator clips (Teishin Electric, model: EA940DT-12)

22. Thermo-hygrometer (INKBIRD, model: IBS-TH1 Plus)

23. Metal scissors (SK11, model: SML-200)

## Software and datasets

1. TsDMMViewer (Ts software, v. 17.1.0)

2. Custom-made image acquisition software developed in LabVIEW

3. Move-tr/2D (Library Ink)

## Procedure

**Figure 1.**
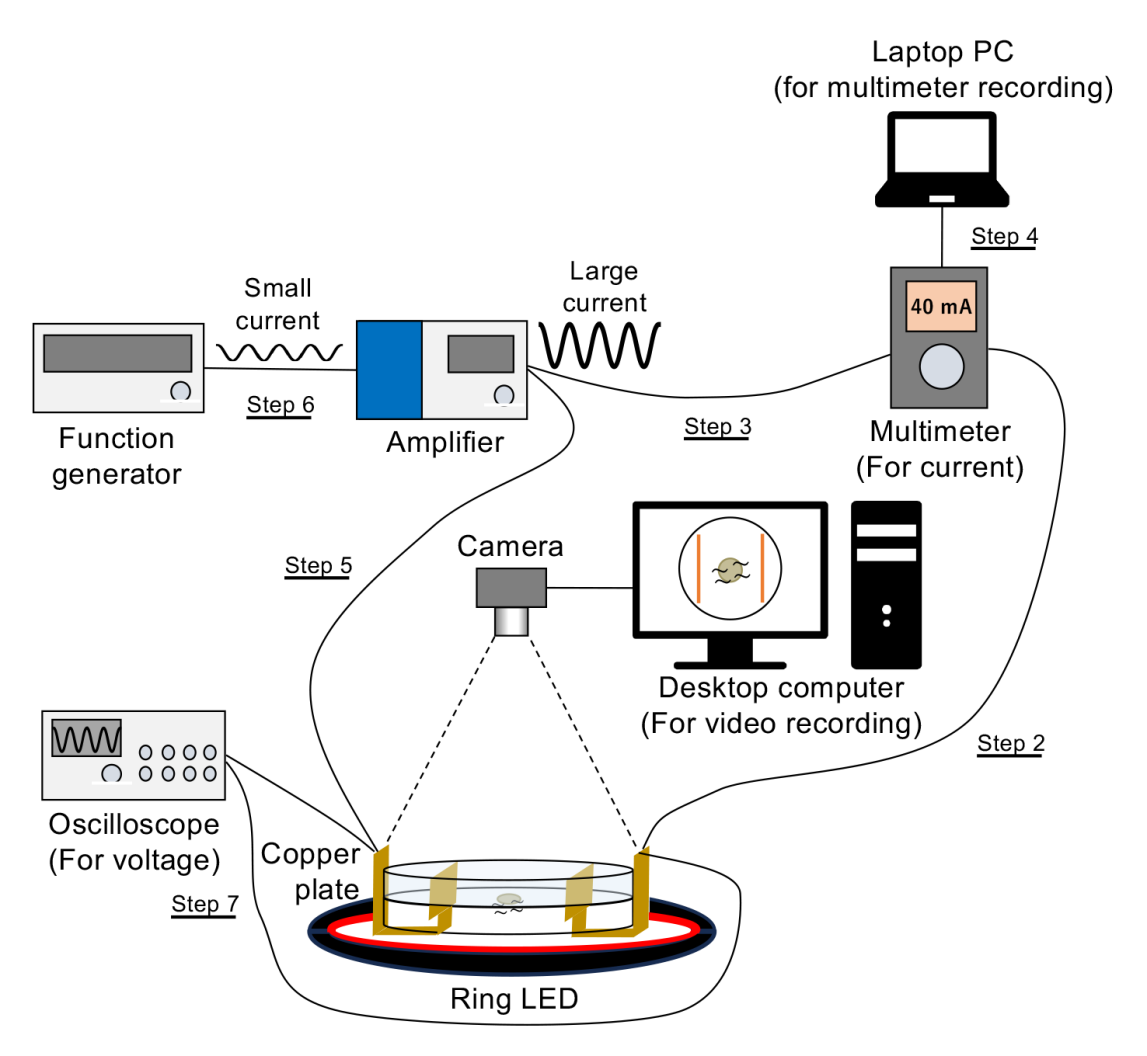
Overview of the experimental system. The system consists of a function generator, amplifier, multimeter, oscilloscope, and video recording setup used to deliver controlled electrical stimulation and record behavioral responses. The function generator produces the input waveform, which is amplified and applied across copper plates. The multimeter records the current, while the oscilloscope monitors the voltage waveform. Worm behavior is recorded using a camera under ring-shaped LED illumination for subsequent tracking and analysis. Step numbers correspond to the Procedure, section A. Adapted from Tee et al. [5] under the terms of the Creative Commons Attribution 4.0 International License.

**A. Apparatus setup (Figure 1)**

**Figure 2.**
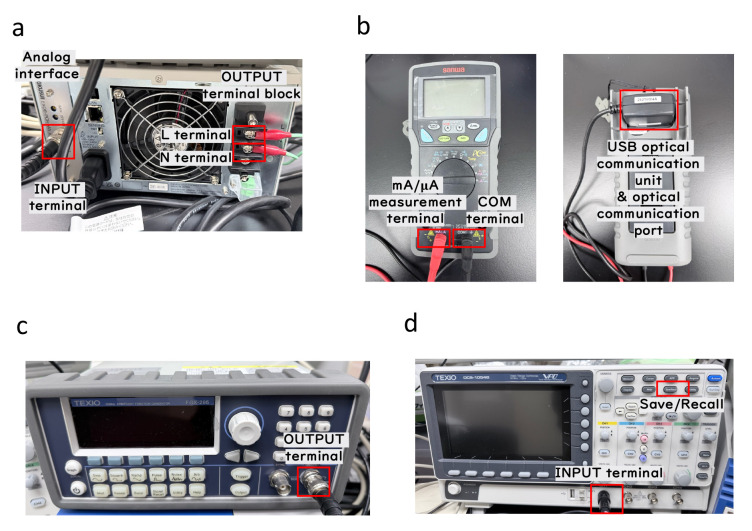
Electrical components and connections used for alternating current (AC) stimulation. (a) Rear panel of the amplifier showing the analog interface, INPUT terminal, and OUTPUT terminal block (L and N terminals). (b) Front (left) and back (right) sides of the multimeter and USB optical communication unit used for current measurement and data acquisition. (c) Function generator used to generate the input waveform. (d) Oscilloscope used to monitor the voltage waveform. Key terminals used for connections are indicated.

1. Amplifier: Insert the analog interface into the optional slot of the amplifier (Figure 2a).

2. Multimeter: Connect the plug of the supplied red cable to the “mA/μA” measurement terminal of the multimeter (Figure 2b, left). To the test pin at the opposite end of this cable, attach a lead wire with alligator clips. The other end of the alligator clip of this wire will be connected to the copper plate.

3. Multimeter: Connect the plug of the supplied black cable to the COM terminal of the multimeter (Figure 2b, left). To the test pin at the opposite end of this cable, attach a lead wire and connect the other end to the N terminal of the OUTPUT terminal block on the amplifier (Figure 2a).

4. Multimeter: Connect the USB optical communication unit (a long cable) to the optical communication port of the multimeter (Figure 2b, right). Connect the USB connector at the opposite end of the cable to the USB port of the laptop PC used for multimeter recording. This connection enables real-time acquisition and recording of current values during the experiment.

5. Amplifier: Connect a lead wire to the L terminal of the OUTPUT terminal block on the amplifier (Figure 2a) and attach another lead wire with an alligator clip to the opposite end of this wire. Connect this alligator clip to the second copper plate.

6. Amplifier: Connect a BNC coaxial cable to the INPUT terminal of the inserted analog interface on the amplifier (Figure 2a) and connect the opposite end of the cable to the OUTPUT terminal of the function generator (Figure 2c). This connection allows the electrical stimulus waveform to be delivered to the amplifier.

7. Oscilloscope: Connect the supplied probe to one of the channel input terminals of the oscilloscope (Figure 2d) and set the probe attenuator on the probe to ×10. Attach a lead wire with an alligator clip to the tip of the probe. Attach another lead wire with an alligator clip to the side ground lead of the probe. The two lead wires will be connected to the copper plates, allowing monitoring of the voltage waveform applied across the plates.

8. Mount the lens on the camera, connect the supplied USB cable to the camera’s USB port, and connect the opposite end of the cable to the USB port of the desktop PC used for image recording.

9. Connect the ring-shaped LED to the LED power supply.

10. Connect all devices to a power strip and turn on the power supply.

**B. Preparation of copper plates**

**Figure 3.**
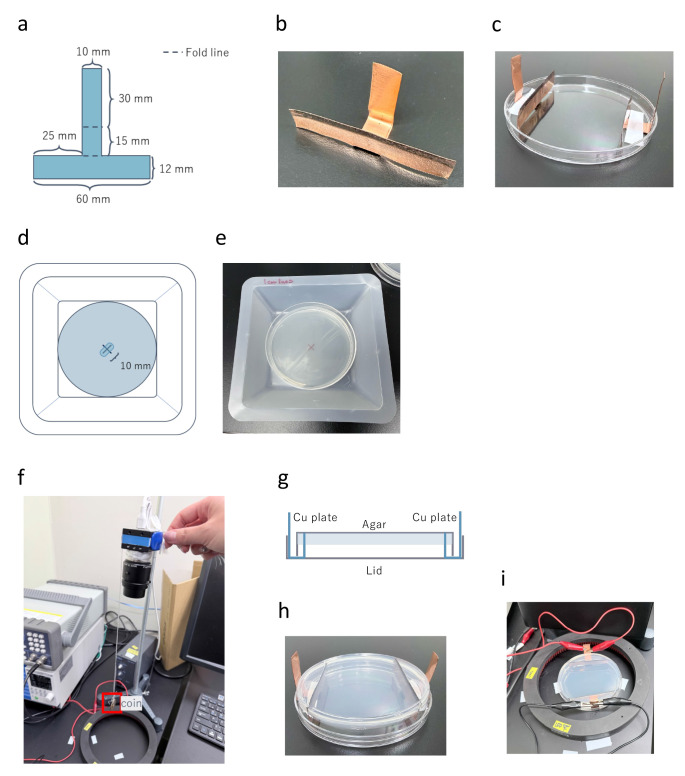
Preparation of copper (Cu) plates and assay setup. (a) Schematic drawing of the copper plate, including dimensions and fold lines. (b) Folded copper plate. (c) Placement of copper plates in a Petri dish. (d, e) Schematic drawing (d) and a photograph (e) of the assay plate placed on a weighing dish used as a positional guide for OP50 dispensing. (f) Setup for camera alignment using a coin suspended on a string (marked with a red square). (g) Cross-sectional schematic showing the insertion of copper plates into the agar. (h, i) Final assembly of the assay plate and wiring configuration (h), and the setup with the lead wires connected (i).

1. Prepare copper plates and cut them using metal scissors, as shown in Figure 3a. Prepare two copper plates.

2. If the horizontal part of the T-shaped copper plate that contacts the agar is somewhat bent or warped, it may not make uniform contact with the agar, resulting in unstable or reduced electrical current flow. Therefore, after cutting the copper plate, check that this horizontal contact surface remains flat. If necessary, gently flatten it before use.

3. Fold the copper plate along the fold line (Figure 3b).

*Note: Replace the copper plates after approximately 60 uses or 1.5 months, whichever comes first. As a guideline, replace the plates when the measured current becomes substantially lower than previous values.*

**C. Worm preparation (day 1)**

Aim: To generate a synchronized population of animals by allowing egg-laying adults to lay eggs for a defined period (6 h).

1. Seed the regular NGM agar plates (1.7% agar in a 60 mm dish) with the OP50 suspension and keep the plates for 2–4 days at room temperature before use (NGM-OP50 plate). This OP50 suspension is prepared by inoculating a single colony into 100 mL of LB medium in a bottle and culturing overnight at 37 °C (without shaking, keeping the cap loosened), followed by storage at 4 °C. Refresh the OP50 stock every 1–2 months.

2. Transfer four gravid adult worms from well-grown cultivated plates onto each NGM-OP50 plate.

3. After transfer, seal each plate with Parafilm and incubate at 19–20 °C for 6 h.

4. After 6 h, remove the plates from the 19–20 °C incubator and remove the Parafilm.

5. Attach a sterile 200 μL pipette tip to the end of the aspirator tubing. Turn on the aspirator, bring the pipette tip close to the worms, and aspirate the worms to remove them from the plate. If worms have burrowed into the agar, insert the tip into the agar and aspirate them.

6. Reseal the plates with Parafilm.

7. For the N2 strain, culture the worms at 19–20 °C and adjust the temperature so that most worms reach the young adult stage at approximately 64 h after this process.

**D. Preparation of killed OP50 (day 4)**

Aim: To provide a thin, uniform bacterial lawn suitable for reliable image analysis. OP50 grown on NGM plates tend to form thick lawns that interfere with the accurate segmentation of worms from the background; therefore, killed OP50 is used.

1. On the previous day (day 3), inoculate OP50 into 100 mL of sterilized LB liquid medium in a flask and culture overnight at 37 °C with shaking at 180 rpm.

2. On day 4, place the NGM buffer and streptomycin solution on ice.

3. Prepare two 50 mL centrifuge tubes and one 1.5 mL microcentrifuge tube.

4. From the flask, decant 45 mL of the culture into each of the two 50 mL centrifuge tubes.

5. Centrifuge the two tubes at 9,000× *g* for 5 min at 4 °C.

6. Discard the supernatant by decanting. Place the inverted tubes onto paper towels to remove residual liquid. Then, close the caps and place the tubes upright in a tube rack.

7. Disrupt the pellets by tapping the tubes by hand. Use a vortex mixer if needed.

8. After both pellets are sufficiently dispersed, add 1 mL of NGM buffer to one of the tubes and suspend the pellet by pipetting.

9. Once the suspension is well mixed, transfer 1 mL of the suspension to the other tube and mix by pipetting. Designate this OP50 preparation as the concentrated OP50.

10. In the 1.5 mL microcentrifuge tube prepared in step D3, mix 900 μL of NGM buffer, 100 μL of the concentrated OP50, and 25 μL of 10 mg/mL streptomycin solution. Mix well.

11. Store this tube on ice.

12. Place an assay plate on a weighing dish marked with an X mark at its center (Figure 3d, e), which serves as a positional guide. Using the X as a reference, dispense 5 μL of the suspension onto the agar surface at one end of a line aligned with the X; take care not to puncture the agar surface during dispensing. Then, tilt the plate and spread the suspension along this line to the opposite end (approximately 10 mm), forming a narrow, bar-shaped OP50 lawn for subsequent placement of worms in section E.

13. Stack the seeded plates with the lids facing upward and leave them at room temperature.

*Note: Complete all of the above steps at least 2 h before starting the assay.*

**E. Electric shock assay (day 4)**

1. Prepare the assay plates from section D and the worms prepared in section C.

2. Transfer 4–6 young adult (YA) worms per plate (depending on experimental conditions) onto the bacterial lawn of each assay plate (made at step D12), cover with a lid, and allow the worms to acclimate at room temperature (22–24 °C). Do not use assay plates that have holes or uneven surfaces. Complete this step at least 1 h before starting the assay.

3. Turn on the two PCs (for the camera and the multimeter), oscilloscope, multimeter, amplifier, function generator, and LED power supply (for the ring-shaped LED).

4. Launch the recording software on the desktop PC used for video acquisition. Any video recording software capable of capturing time-lapse images at ≥2 frames per second can be used. In our experiments, we used custom software developed in LabVIEW.

5. Place an assay plate containing worms (used only for setup, not for the assay) inside the ring-shaped LED. To ensure that the camera is oriented vertically to the bench, suspend a coin attached to a string above the field of view. Adjust the camera tilt while observing the coin until it appears stationary and centered in the image, indicating proper vertical alignment (Figure 3f).

6. While viewing the PC screen, adjust the focus and aperture of the camera to ensure clear visualization of worms for subsequent tracking analysis. Remove the plate once the adjustment is done.

7. On the laptop PC used for multimeter recording, launch TsDMMViewer and select *CONNECT* in the upper-left area of the interface to connect to the multimeter.

8. Insert a USB storage into the oscilloscope.

9. Set the vertical scale of the oscilloscope to 5 V and the horizontal time scale to 50 s.

10. Set the function generator to 450 mVpp (30 V) or 1,050 mVpp (75 V) at 4 Hz.

11. After setting the parameters, press the output button on the function generator to deliver the signal to the amplifier.

12. Attach the copper plates prepared in section B to the lid of a clean plate using vinyl tape and place the lid with the inner surface facing upward (Figure 3c, g, h).

13. Place an assay plate without worms onto the lid with copper plates and press it down until the plate firmly adheres to the lid.

14. Place the lid and plate inside the ring-shaped LED and attach the alligator clips to the copper plates (Figure 3i).

15. Start recording on the oscilloscope and multimeter.

16. Press the output button on the amplifier to apply current and check the readings on the multimeter and oscilloscope (approximately 80 mA at 30 V and 200 mA at 75 V).

17. If the expected signals are observed in the multimeter and oscilloscope, stop the oscilloscope recording and remove the plate without worms.

18. Turn off the room lights for clear image acquisition with the video.

19. Set the assay plate containing worms, prepared in step E2, in the same manner as described in steps E13–14.

20. Operate the devices according to the on/off timeline shown in Figure 4a. When the voltage is applied, N2 (wild-type) worms should exhibit an increase in locomotor activity. At moderate stimulation (e.g., 30 V), this increase is typically visible immediately, whereas at higher stimulation (e.g., 75 V), the response may develop with a short delay (Figure 4b).

**Figure 4.**
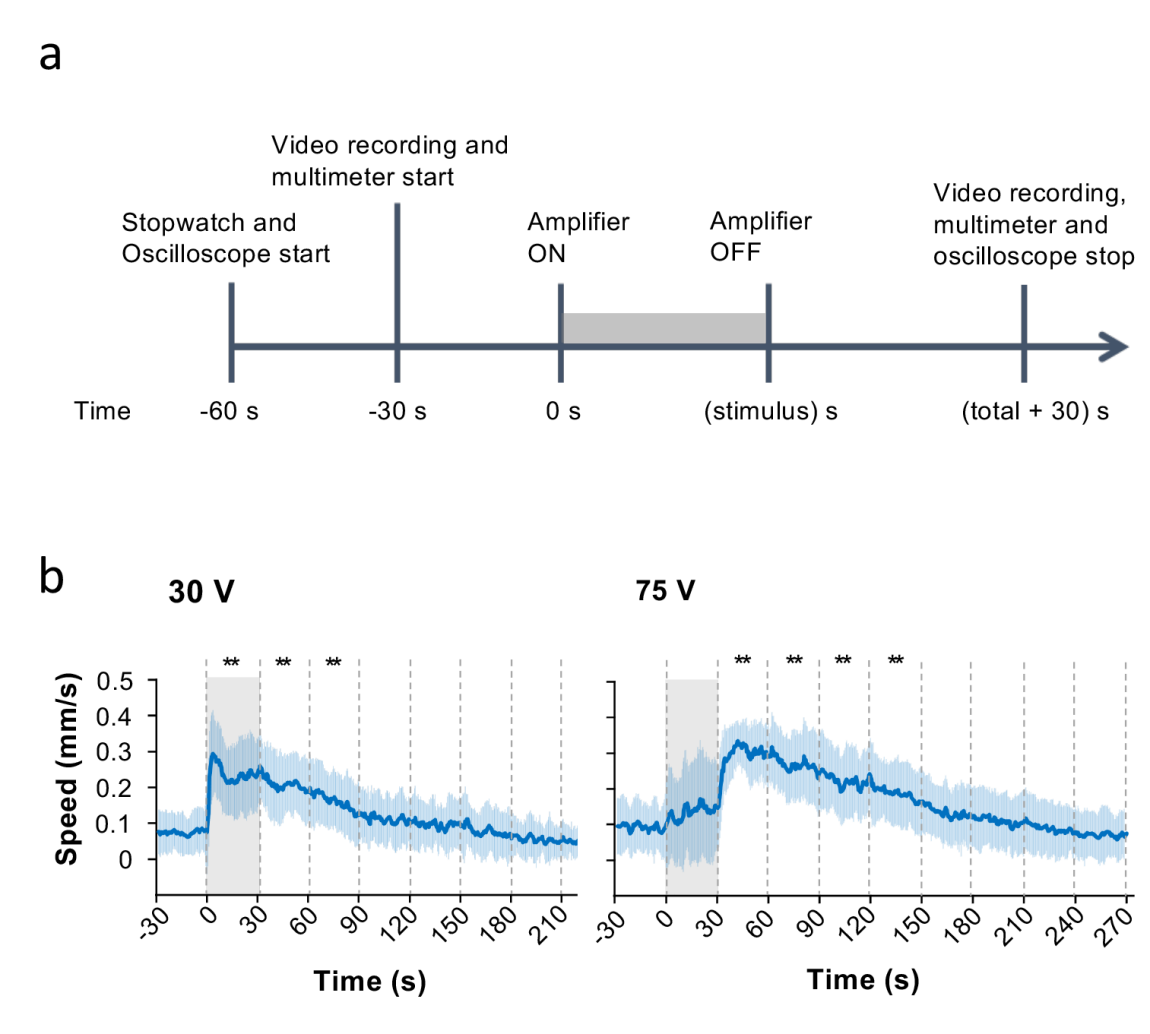
Experimental timeline and representative behavioral responses. (a) Timeline of the recording and stimulation protocol. Video recording begins prior to stimulation, followed by application of alternating current (AC) stimulation and subsequent recording of post-stimulus behavior. (b) Representative locomotor responses of N2 (wild-type) worms under different stimulation conditions (30 V, 240 s; 75 V, 30 s). Gray shading indicates the stimulation period. Statistical values were calculated using the Kruskal–Wallis test with Bonferroni correction for the differences from the average speed before the stimulation. **P < 0.001. Adapted from Tee et al. [5] under the terms of the Creative Commons Attribution 4.0 International License.

21. After completing the measurement, press the *Save/Recall* button in the upper-left corner of the oscilloscope and save the images and data (Figure 2d): *Save image* → *Save; Save waveform* → *To file* → *Detail CSV* → *Save*.

22. Next, click the *Save* button in the multimeter software on the laptop PC and save the data in the folder labeled with the date of acquisition.

23. After saving, click the *Clear* button to reset the software.

24. Remove the electrodes from the plate and wipe them with a Kimwipe.

25. Because the lid may be fogged, gently wipe it with another Kimwipe moistened with water. Replace the lid with a new one if it becomes dirty.

26. Attach the electrodes to the next plate.

27. Repeat steps E19–26 for subsequent measurements.

## Data analysis

For each experimental condition, we recommend analyzing 20–30 animals per condition to ensure robust estimation of locomotor responses. All the animals were included in the analysis without exclusion. Experiments were typically performed using ≥2 plates per day and repeated on 2–4 days to confirm reproducibility.

Worm trajectories are defined as the time series of centroid positions of individual worms. These positions were extracted from recorded videos using tracking software. In our experiments, we used Move-tr/2D (Library Inc., Japan); similar results should be obtained using other tracking software capable of extracting centroid positions from time-lapse images.

Individual centroids were tracked, and locomotor speed was calculated from the exported xy coordinate data. The frame rate was set to *f* = 2 frames per second (fps), and ∆*t* = 1/*f*. The displacement (*d*_i_) and velocity (*v*_i_) were calculated as follows:

$$d_{i}=\sqrt{{\left(x_{i}-x_{i-1}\right)}^{2}+{\left(y_{i}-y_{i-1}\right)}^{2}}$$

$$v_{i}=\frac{d_{i}}{∆t}$$

Velocity traces were smoothed using a three-point moving median. Median velocities before stimulation (-30 to 0 s) and after stimulation (every 30 s) were calculated and compared using the Wilcoxon signed-rank test followed by Bonferroni correction.

Data inclusion and exclusion: All animals within the field of view were included in the analysis, including those that exhibited little or no movement. Datasets were excluded only when technical issues were identified. In particular, recordings in which the measured current was substantially reduced (e.g., <10% of the typical value), indicating improper insertion or contact of the copper plates, were excluded from analysis.

## Validation of protocol

This protocol has been used and validated in the following research article:

• Tee et al. [5]. Electric shock causes a fleeing-like persistent behavioral response in the nematode *Caenorhabditis elegans*. GENETICS (Figures 1–4). https://doi.org/10.1093/genetics/iyad148

## General notes and troubleshooting

**General notes**

Environmental factors such as temperature, humidity, and culture conditions may influence the behavioral response. In our experimental setup, measurements were performed under controlled room conditions (22–24 °C, 30%–55% humidity) to minimize variability. Animals were restricted to a defined developmental stage (young adults) to further reduce variability across individuals. Factors such as age and sex may influence behavioral responses; however, in this protocol, we used synchronized young adult hermaphrodites under standardized conditions. Under these conditions, the assay was robust and yielded consistent results across experiments. To ensure reproducibility, experiments were performed using multiple plates and repeated on different days. Recommended sample sizes and basic inclusion criteria are described in the Data analysis section.

Limitations: This protocol relies primarily on locomotor speed as a behavioral readout and therefore may not capture other aspects of behavior, such as posture, turning, or directionality. These limitations should be considered when interpreting results and designing experiments.

**Troubleshooting**

**Problem 1:** No or small electrical current flows.

Possible cause: The copper plates are not properly inserted into the agar.

Solution: Check the size of the copper plates and confirm that there is no gap between the copper plates and the agar.

**Problem 2:** The electrical current is unstable.

Possible causes: The copper plates are excessively contaminated, or the composition of the NGM plates is incorrect.

Solution: Prepare new copper plates or remake the NGM plates.
